# Development and validation of the Axillary Sweating Daily Diary: a patient-reported outcome measure to assess axillary sweating severity

**DOI:** 10.1186/s41687-019-0148-8

**Published:** 2019-09-05

**Authors:** L. M. Nelson, D. DiBenedetti, D. M. Pariser, D. A. Glaser, A. A. Hebert, H. Hofland, J. Drew, D. Ingolia, K. K. Gillard, S. Fehnel

**Affiliations:** 1 RTI Health Solutions, 200 Park Offices Drive, Research Triangle Park, NC 27709 USA; 2 Eastern Virginia Medical School Department of Dermatology and Virginia Clinical Research, Inc, 6160 Kempsville Road Suite 200A, Norfolk, VA 23452 USA; 3 Department of Dermatology, Saint Louis University, 1755 S. Grand Blvd, St. Louis, MO 63104 USA; 4 Department of Dermatology, UTHealth McGovern Medical School at Houston, 6655 Travis, Suite 980, Houston, TX 77030 USA; 5 Dermira, Inc, 275 Middlefield Road, Suite 150, Menlo Park, CA CA 94025 USA

**Keywords:** Hyperhidrosis, Sweating severity, Patient reported outcome, Axillary sweating daily diary (ASDD)

## Abstract

**Background:**

Hyperhidrosis is estimated to affect ~ 4.8% of the US population, and most patients experience a negative psychological impact. Here, we describe development and psychometric evaluation of a patient-reported outcome (PRO) measure to assess severity of axillary hyperhidrosis in clinical trials that meets current U.S. regulatory standards to support product approvals.

**Methods:**

Three rounds of hybrid concept-elicitation/cognitive-debriefing qualitative interviews were conducted in adults with clinician-diagnosed primary axillary hyperhidrosis, followed by similar interviews in children/adolescents. The draft measure included diary items for presence, severity, impact and bothersomeness (basis of the Axillary Sweating Daily Diary [ASDD]), exploratory weekly impact items, and a single-item Patient Global Impression of Change (PGIC). Phase 2 (adults only) and phase 3 (adults and children ≥9 years) clinical trial data were utilized to evaluate measurement properties of the resulting draft measure: floor/ceiling effects, nonresponse bias, test-retest reliability, construct validity, and responsiveness were assessed. The primary concept of interest was axillary sweating severity (ASDD Item 2); however, additional supportive concepts were explored to allow for development of a comprehensive hyperhidrosis measure.

**Results:**

Twenty-nine patient interviews were conducted (*N* = 21 adult and *N* = 8 children/adolescents), resulting in the ASDD (4 items, patients ≥16y) and child-specific ASDD-C (2 items ≥9y to <16y), as well as 6 Weekly Impact items and the PGIC (patients ≥16y). No floor/ceiling effects or response biases were identified. Consistency between hypothesized and observed correlation patterns between ASDD/ASDD-C items and other efficacy measures supported construct validity. Intraclass correlation coefficients supported test-retest reliability (0.91–0.93; Item 2). Large effect sizes (− 2.2 to − 2.4) demonstrated that the ASDD/ASDD-C Item 2 could detect changes in hyperhidrosis severity, supporting the measure’s responsiveness. Patients perceiving a moderate improvement in symptoms on the PGIC experienced an average 3.8-point improvement on ASDD axillary sweating severity (Item 2); thus, a 4-point responder threshold was defined as a clinically meaningful change.

**Conclusions:**

Qualitative and quantitative evidence support the reliability and validity of the ASDD/ASDD-C and its use in the clinical evaluation of axillary hyperhidrosis treatments. Further evaluation of this measure in future research studies is warranted to demonstrate consistent performance across different axillary hyperhidrosis populations and in different study contexts.

## Background

Hyperhidrosis (HH) is a condition in which sweat production exceeds that which is physiologically necessary to maintain thermal homeostasis [1]. This burdensome condition is estimated to affect up to 4.8% of the U.S. population, or 15.3 million Americans, and causes substantial impairment in patient daily life, as approximately three-quarters of patients report negative impacts on their social life, sense of well-being, and/or mental health [2].

A number of tools have been utilized to measure patient-reported severity, impact, and bother associated with hyperhidrosis [3]. Generic health-related quality of life (HRQOL) measures such as the Short Form Health Surveys (SF-36 and SF-12) and the Nottingham Health Profile (NHP) have been used [4, 5]; however, these cover broader constructs of health, do not necessarily reflect areas of functioning that are particularly relevant to patients with hyperhidrosis, and lack sensitivity needed to discern clinically meaningful treatment benefit [6]. Similarly, dermatology-specific measures (Skindex, Dermatology Life Quality Index [DLQI]) have been administered to patients with hyperhidrosis but were not developed to capture all relevant hyperhidrosis-specific concepts [7–9]. The most commonly used hyperhidrosis-specific scale is the Hyperhidrosis Disease Severity Scale (HDSS). Although this scale has been broadly adopted in clinical practice to evaluate disease severity, it combines two distinct concepts in the same item (ie, tolerability and interference with daily activities), thus limiting interpretation of study results. In addition, patient input was not solicited during the development of the HDSS [10, 11]. Other disease-specific measures include the Hyperhidrosis Impact Questionnaire (HHIQ) [12], the Amir-de Campos Clinical Protocol for QoL [13, 14], and the Keller scale [15]. The Hyperhidrosis Quality of Life Index (HidroQOL©) is a more recently developed measure; however, at the time this research was designed and conducted, it was not publicly available and its measurement properties had not been evaluated in an interventional trial setting [16, 17].

Although the instruments described above have been used to better characterize patients with hyperhidrosis, it is unclear whether any were developed to support product approvals in accordance with most recent U.S. regulatory standards for PRO measures (ie, for inclusion as key endpoint(s) in a clinical trial) [18]. This prompted the development of a new PRO to measure hyperhidrosis treatment benefit to meet U.S. registration needs, and to more broadly understand the impact of hyperhidrosis on patients’ lives.

Here, we describe the development, features, and validation of the Axillary Sweating Daily Diary (ASDD) and Axillary Sweating Daily Diary-Children (ASDD-C), with a focus on the psychometric evaluation of axillary sweating severity item (Item 2).

## Methods

### Instrument development

**Fig. 1:**
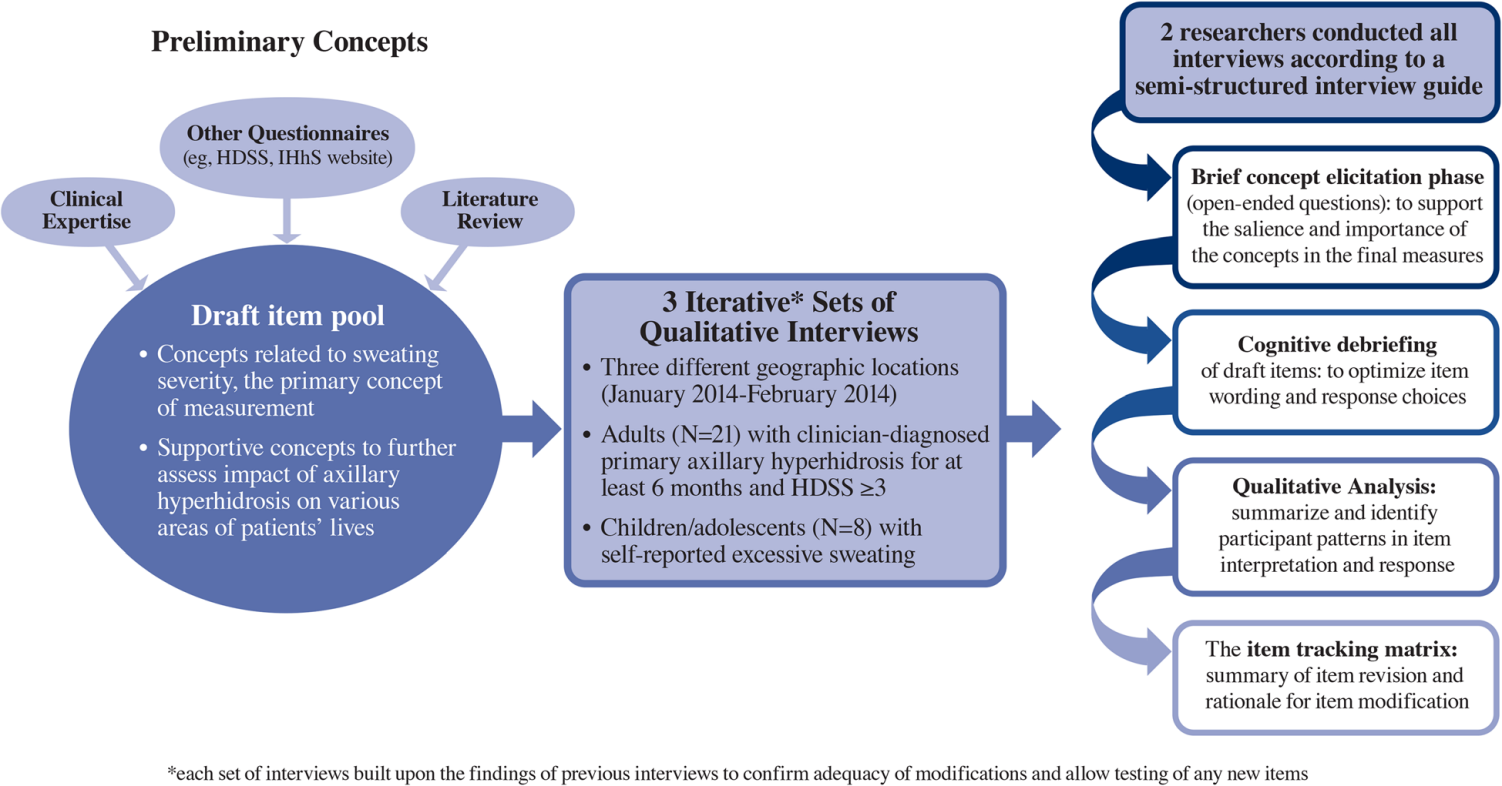
Overview of the Instrument Development Process. ASDD, Axillary Sweating Daily Diary; HDSS, Hyperhidrosis Disease Severity Scale; IHhS, International Hyperhidrosis Society

A brief overview of the instrument development process is detailed in Fig. 1. Specifically, one-on-one hybrid concept-elicitation/cognitive-debriefing qualitative interviews were conducted with patients ≥18 years with clinician-diagnosed primary axillary hyperhidrosis (*N* = 21) in 3 different geographic locations between January 2014 and February 2014. Interview results were qualitatively analyzed in three sets, where each set of interviews built upon the findings of previous interviews to confirm the adequacy of modifications and allow testing of any new items. Consistent with the inclusion criteria for the initial dose-finding trials, all participants were required to exhibit hyperhidrosis symptomology for at least 6 months, as well as report an HDSS score of 3 or 4, indicating “barely tolerable” or “intolerable” sweating and sweating “frequently” or “always” interfering with daily activities. Preliminary concepts were selected for inclusion in a draft item pool based on clinical expertise, review of the literature, and review of other questionnaires (eg, HDSS, questions on the International Hyperhidrosis Society website [www.sweathelp.org]). Given that the primary concept of measurement, sweating severity, was both straightforward and clearly important within the context of hyperhidrosis, draft items addressing this concept were developed prior to patient engagement and were comprised of daily diary items covering concepts pertaining to presence, severity, impact and bother of axillary hyperhidrosis (ultimately referred to as the ASDD). Additional supportive concepts were selected for inclusion in a preliminary item pool to further explore the impact of axillary hyperhidrosis on various areas of patients’ lives (ultimately referred to as the Weekly Impact Items). A Patient Global Impression of Change (PGIC) item was also included to assess patient-perceived changes in underarm sweating and facilitate the interpretation of change scores on the diary items. Multiple questions were drafted for each concept so variable wording and response scales could be tested during patient interviews. All interviews were conducted by two experienced qualitative researchers according to a semi-structured interview guide created collaboratively by the developers. Each interview began with open-ended concept elicitation. Participants were asked to describe their experiences with axillary sweating, including variations in severity, the extent and situations in which they were bothered by their sweating, and how their lives were impacted by hyperhidrosis. Following the concept elicitation phase, the same patients were then asked to provide feedback on the draft items via paper forms during a cognitive debriefing phase to support the salience and importance of the concepts, as well as to optimize item wording and response options. Specifically, participants were asked to respond to each item while thinking aloud. Interviews were reviewed individually and compared to other interviews in order to summarize and identify participant patterns in item interpretation and response. An item tracking matrix was created, which included any revisions made to an item and a rationale for the item modification. Additional follow-up questions were also posed by the interviewers to further elucidate comprehension and response processes. If concept saturation was not reached following qualitative data analysis based on the findings during each set of interviews, additional interviews were to be conducted with new patients.

Following the decision to include individuals as young as 9 years of age in a future clinical trial program, a similar set of qualitative interviews was conducted with children and adolescents < 18 years with self-reported excessive sweating (*N* = 8) to evaluate whether the new measure would be relevant for children ≥9 years of age. While the sample size was deemed adequate to achieve concept saturation, if new concepts had been generated during these interviews, testing with additional pediatric patients would have been conducted.

### Psychometric evaluation

#### Phase 2 and 3 study design and analysis

To further support use of the ASDD/ASDD-C axillary sweating severity item (Item 2) in clinical trials evaluating the efficacy of new treatments for hyperhidrosis, the measurement properties of ASDD Item 2 were evaluated using data from a 4-week phase 2 trial of glycopyrronium tosylate (GT) in patients ≥18 years of age (study DRM04-HH02, NCT02129660; *N* = 102) [19]. Phase 2 data were also used to establish basic psychometric properties of ASDD impact and bothersomeness items (Items 3 and 4). Weekly Impact items, PGIC, HDSS, and gravimetric data were also collected as study outcomes.

Additional psychometric evaluation of ASDD/ASDD-C Item 2 was subsequently conducted based on pooled data from two, 4-week phase 3 trials of GT, ATMOS-1 (DRM04-HH04, NCT02530281; *N* = 344; sites in the U.S. and Germany) and ATMOS-2 (DRM04-HH05, NCT02530294; *N* = 353; U.S. sites only) [20]. Study designs and inclusion criteria were similar for the phase 2 and phase 3 studies; the phase 3 study also included the Dermatology Life Quality Index (DLQI) and Children’s Dermatology Life Quality Index (C-DLQI, for ages 4 to 16 years) in addition to the other outcomes collected and described above for phase 2. The DLQI/C-DLQI is a dermatology-specific, health-related quality of life questionnaire developed to understand the impacts of skin conditions on patients’ lives [9, 21]. Eligible patients were ≥ 18 years of age for phase 2 and ≥ 9 years of age for the phase 3 studies; had a diagnosis of primary axillary hyperhidrosis for ≥6 months; had HDSS grade 3 or 4; had sweat production ≥50 mg/axilla/5 min while at rest at room temperature; and for phase 3 studies, had weekly average ASDD Item 2 scores ≥4.

PRO measures were completed by patients using an electronic tablet device provided to them during the study period as follows: for patients ≥16 years of age, the ASDD was completed daily (before bed) for 4 to 7 days during screening (7 to 10 days prior to randomization) and during treatment; in the phase 3 studies, patients < 16 years completed the ASDD-C items (Items 1 and 2 only) according to the same schedule (phase 3 only). Patients ≥16 years of age additionally completed two other measures developed alongside the ASDD, namely the Weekly Impact items (Table 1) and the single-item PGIC (Table 1)**.** These 3 measures (the ASDD, the Weekly Impact items, and the PGIC) are collectively referred to in Table 1 as the Axillary Hyperhidrosis Patient Measures (AHPM). The HDSS and Weekly Impact items were completed at Baseline and each week during the treatment period; the DLQI (phase 3 only) and PGIC was completed at Week 4 or at the end of treatment (ie, early termination). The ASDD/ASDD-C, Weekly Impact items, and PGIC were completed via electronic data capture; the HDSS and DLQI/C-DLQI (phase 3 only) were completed weekly on paper. Sweat production, assessed gravimetrically, was measured for the right and left axilla at Baseline and each weekly clinic visit.

**Table 1:** Axillary Hyperhidrosis Patient Measures (AHPM)

Axillary Sweating Daily Diary (ASDD)^a^	
*Instructions: The questions in the diary are designed to measure the severity and impact of any underarm sweating you have experienced within the previous 24-h period, including nighttime hours. While you may also experience sweating in other locations on your body, please be sure to think only about your underarm sweating when answering these questions.* *Please complete the diary each evening before you go to sleep.*	
Item 1 [Gatekeeper]	During the past 24 h, did you have any underarm sweating? *Yes/No* When Item 1 is answered “no,” Item 2 is skipped and scored as zero
Item 2	During the past 24 h, how would you rate your underarm sweating at its worst? *0 (no sweating at all) to 10 (worst possible sweating)*
Item 3	During the past 24 h, to what extent did your underarm sweating impact your activities? *0 (not at all), 1 (a little bit), 2 (a moderate amount), 3 (a great deal), 4 (an extreme amount)*
Item 4	During the past 24 h, how bothered were you by your underarm sweating? *0 (not at all bothered), 1 (a little bothered), 2 (moderately bothered), 3 (very bothered), 4 (extremely bothered)*
Axillary Sweating Daily Diary-Children (ASDD-C)^b^	
*Instructions: These questions measure how bad your underarm sweating was last night and today. Please think only about your underarm sweating when answering these questions.* *Please complete these questions each night before you go to sleep.*	
Item 1 [Gatekeeper]	Thinking about last night and today, did you have any underarm sweating? *Yes/No* When Item 1 is answered “no,” Item 2 is skipped and scored as zero
Item 2	Thinking about last night and today, how bad was your underarm sweating? *0 (no sweating at all) to 10 (worst possible sweating)*
Weekly Impact Items^a^	
*Instructions: Please respond “Yes” or “No” to each of the following questions.*	
a. During the past 7 days, did you ever have to change your shirt during the day because of your underarm sweating?	*Yes/No*
b. During the past 7 days, did you ever have to take more than 1 shower or bath a day because of your underarm sweating?	*Yes/No*
c. During the past 7 days, did you ever feel less confident in yourself because of your underarm sweating?	*Yes/No*
d. During the past 7 days, did you ever feel embarrassed by your underarm sweating?	*Yes/No*
e. During the past 7 days, did you ever avoid interactions with other people because of your underarm sweating?	*Yes/No*
f. During the past 7 days, did your underarm sweating ever keep you from doing an activity you wanted or needed to do?	*Yes/No*
Patient Global Impression of Change (PGIC) Item^a^	
Overall, how would you rate your underarm sweating now as compared to before starting the study treatment?	
1 (much better), 2 (moderately better), 3 (a little better), 4 (no difference), 5 (a little worse), 6 (moderately worse), 7 (much worse)	

^a^ For use in patients ≥16 years of age

^b^ For use in patients ≥9 to < 16 years of age

Copyright© Dermira, Inc. 2017

ASDD/ASDD-C items were scored as a weekly average of daily responses; at least 4 days of daily data were required for analysis. Responses to the 6 Weekly Impact items were summed such that a response of “Yes” to any of the items resulted in a score of 1 per item. This was referred to as the Weekly Impact summary (possible score range of 0 to 6) and used in exploratory analyses. All statistical tests were two-tailed using a type I error rate of 5% (alpha = 0.05). Rather than relying on statistical significance alone, the interpretation of effect size estimates and patterns of results were emphasized. Observed values were used for all calculations for the phase 2 study. For ASDD Item 2 in the phase 3 studies, the weekly mean from Markov Chain Monte Carlo (MCMC) imputation was used; observed values were used for ASDD Items 3 & 4 as well as the PGIC. For binary response data (i.e., ASDD/ASDD-C Item 1, Weekly Impact items), missing values were considered a “No” response.

#### Evaluation of measurement properties

Potential floor and ceiling effects (approximately double the expected proportion given equal distribution across response options or ≥ 20% of sample responding at either extreme of the scale), were evaluated based on responses during screening (Baseline) using both summary statistics and graphical techniques. Test-retest reliability for Item 2, 3, and 4 was evaluated to assess each item’s stability through the computation of intraclass correlation coefficients (ICCs) between Week 3 and Week 4, using a threshold of ≥0.70 [22]. Week 3 and Week 4 were chosen as they represented time points at which symptoms were considered to be most stable following initiation of treatment.

Construct validity was evaluated at Week 4 based on correlations between ASDD/ASDD-C Item 2 gravimetric measurements and established PRO measures (HDSS and DLQI). Week 4 correlations were also explored between ASDD/ASDD-C Item 2 and ASDD Items 3 and 4, the Weekly Impact items, and the PGIC [23]. Correlations ≥0.50 were considered strong, ≥0.30 to < 0.50 moderate, and < 0.30 weak [24].Correlations with items addressing related constructs (Items 3 and 4) were hypothesized to yield larger correlations as compared with those computed between items addressing more disparate constructs: given that sweating can be episodic and can vary based on time of day, emotional stimuli, and/or daily activities [25], weak positive correlations were expected between ASDD/ASDD-C Item 2 scores and gravimetric measurements. Weak to moderate correlations were expected between DLQI and ASDD/ASDD-C Item 2, as DLQI may capture some, but not all, hyperhidrosis-specific issues. The HDSS was expected to correlate moderately to strongly with ASDD/ASDD-C Item 2 as both items are disease-specific, although the correlations could be mitigated due to the limitations in HDSS described above.

Known-groups validity (the ability of the measure to discriminate between clinically distinct subgroups) was evaluated at Week 4 based on two-sample t-tests comparing differences in scores on ASDD/ASDD-C Item 2 between subgroups predefined based on HDSS grades (score of 1 vs score of 3 or 4), DLQI score ranges (range of 0–10 and 0–12 for no to moderate impact for DLQI and CDLQI, respectively, and range of 21–30 and 19–20 for large to extremely large impact for DLQI and CDLQI, respectively) [9, 21, 26, 27], and gravimetrically-measured sweat production (1st vs 4th distribution quartiles).

Responsiveness was evaluated through effect size computation between Baseline and Week 4 to evaluate the ability of the ASDD/ASDD-C items to detect change. A responder definition to establish a threshold for a magnitude of within-person change considered to be clinically meaningful was determined based on methods outlined in the FDA PRO guidance [18], which recommends the use of self-reported retrospective measures of change as external anchors for the estimation of meaningful change. Such an anchor must be both a valid measure of change and easier to interpret than the PRO measure [28]. Thus, the PGIC was utilized as the predefined anchor. Specifically, responders were defined as patients who rated their underarm sweating as “moderately better” on the PGIC compared with the start of study treatment. Exploratory within-person changes in HDSS scores were also calculated against PGIC responses. Finally, although PGIC measures are widely accepted as an appropriate anchor [28], exploratory within-person change scores on the ASDD were calculated across different magnitudes of change in HDSS scores.

## Results

### Instrument development

**Fig. 2:**
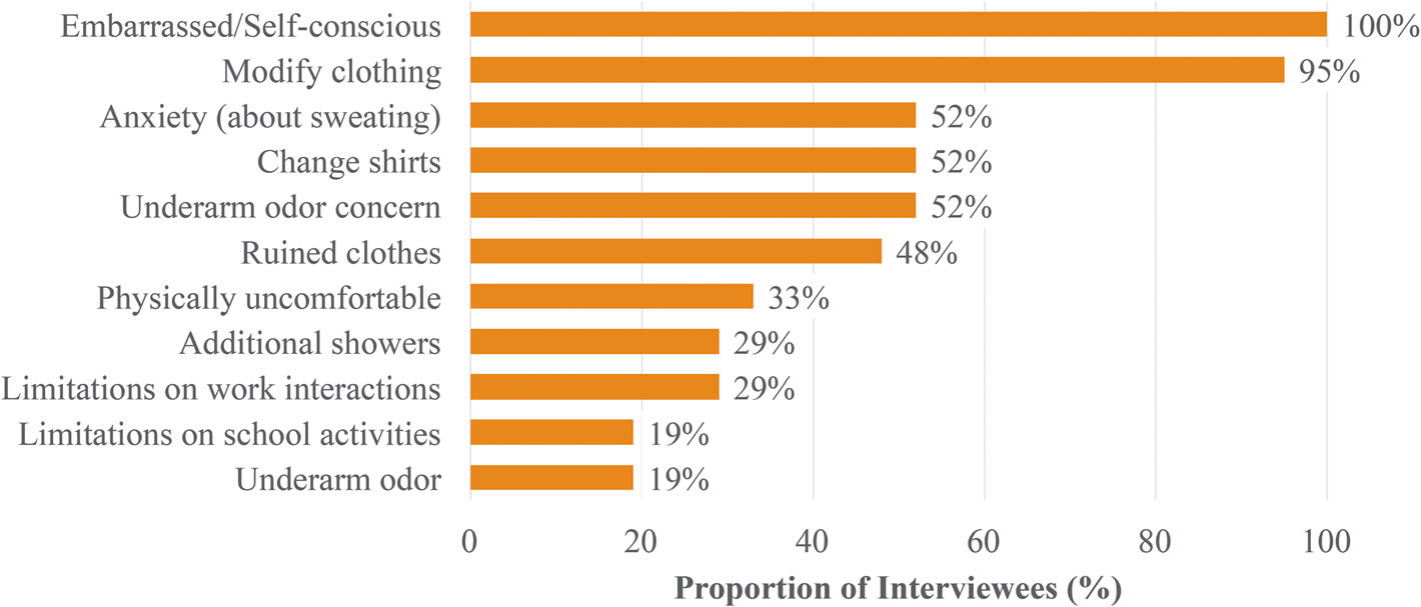
Areas of bother and impact associated with axillary hyperhidrosis (Adult Patient Interviews; *N* = 21)

The cohort of adult interviewees was predominantly white (76%) and female (76%) (Table 2). In general, adult interviewees reported that the most relevant diary items intended for daily administration as part of the ASDD included those related to the presence, severity, overall impact, and bother associated with hyperhidrosis. Participants noted that the 24-h reference period was appropriate. Several of the items originally proposed for inclusion in the ASDD were considered to be salient to some patients at some points in time (interference with activities, need for additional showers/baths, and need for additional shirt changes); thus, they were better suited for inclusion in the Weekly Impact items (7-day recall) and tested as such in subsequent rounds of interviews. Additional concepts tested and retained as a component of the Weekly Impact items included avoiding social interactions, confidence and embarrassment. Of note, all adult interviewees reported embarrassment due to their axillary hyperhidrosis and nearly all (95%) modified clothing choices due to their symptoms (Fig. 2).

**Table 2:** Characteristics of Interviewees Reported at Screening

Characteristic	Adults (*N* = 21)	Pediatric (*N* = 8)
Excessive sweating locations, n (%)^a^		
Underarm	21 (100)	8 (100)
Hands	6 (29)	4 (50)
Feet	6 (29)	3 (37.5)
Head	6 (29)	5 (62.5)
Back	4 (19)	5 (62.5)
Groin	0 (0)	2 (25)
Sex, n (%)		
Male	5 (24)	4 (50)
Female	16 76)	4 (50)
Age (years), mean (range)	36 (18–57)	13.1 (10–17)
Race, n (%)		
White	16 (76)	3 (37.5)
Black	1 (5)	0 (0)
Hispanic	4 (19)	3 (37.5)
Other	0 (0)	2 (25.0)
Current medication use for any^b^ excessive sweating, n (%)^c^		
Over-the-counter	12 (57)	7 (87.5)
Prescription	10 (48)	1 (12.5)
Previous medication use for any^b^ excessive sweating, n (%)^c^		
Over-the-counter	20 (95)	7 (87.5)
Prescription	16 (76)	1 (12.5)

^a^ Total sum exceeds 100%, as participants could report excessive sweating in multiple locations

^b^ Excessive sweating refers to any area of excessive sweating (ie, not restricted to axillary sweating)

^c^ Total sum exceeds 100%, as participants could report concurrent use of more than one prescription and/or over-the-counter medication

Minor modifications to item wording were incorporated as needed following each round of interviews to ensure content was clear, comprehensive, and easy to understand. Concepts pertaining to underarm odor severity and interference with sleep were not generally endorsed by patients and deleted, as patients indicated that it was the anxiety about having odor that was most impactful and not necessarily the odor itself, and that hyperhidrosis symptoms typically occurred during the day and not while sleeping. The PGIC was also tested, with no revisions made in all three rounds of interviews as patients found the item clear and easy to answer in the context of receiving hyperhidrosis treatments. No new concepts were introduced as part of the concept elicitation portion of the interviews, and concept saturation was reached across the three sets of qualitative interviews. Thus, no additional patient interviews were required for further input.

The level of bother was consistent across both adult and pediatric samples. The most commonly reported impacts of hyperhidrosis in both populations included feeling embarrassed/self-conscious, modifying types of clothes worn, concern about underarm odor, changing shirts throughout the day, anxiety about sweating, ruined clothes, and physically uncomfortable due to excessive underarm wetness. Adults reported work-related impairments such as limiting interactions with work colleagues and nonmandatory work activities, consistent with the school-related impairments such as avoiding school-based activities and difficulty studying reported by the pediatric sample.

Like the adult interviewees, pediatric interviewees (*N* = 8) reported being bothered or burdened by the following: feeling embarrassed; sweating a lot more than others in similar circumstances; feeling sweat dripping down sides of the body; needing to cover up their sweating with hair or jackets/sweatshirts, even in hot weather; needing to “air their armpits out”; feeling like they have a bad smell; feeling sticky; not liking the wet feeling; and feeling itchy. Of note, adults did not report feeling itchy in conjunction with their excessive sweating. The three 17-year-old participants reported that their excessive underarm sweating began between during 6th or 8th grade. Three other participants (aged 10, 11, and 13 years) reported that their excessive sweating began around 4th grade and was most noticeable during sports or other physical activities. The remaining participants (aged 10 and 11 years) did not know when their excessive sweating began, stating that it was likely around age 4 to 5 years.

All 17-year-olds (*N* = 3) found the instructions and recall period on the ASDD clear and easy to understand. In contrast, 4 of the 5 younger participants (aged 10 to 13 years) had difficulty reading and understanding the instructions and/or understanding the 24-h recall period. Based on these findings, the instructions and recall periods used in the ASDD-C were modified for patients < 16 years of age, as reflected in Table 1. Many of the younger pediatric interviewees had difficulty understanding and answering the ASDD items addressing the impact and burden of sweating (Items 3 and 4, respectively) even after modification; therefore, these items were eliminated from the ASDD-C. As many also had difficulty with the extended recall periods for the Weekly Impact items and the PGIC, these items were not administered to patients < 16 years of age in the phase 3 trials.

The PRO measures resulting from the qualitative phase of research are shown in Table 1. Item 1 is a gating question followed by Item 2, which queries level of axillary sweating severity using a 0-to-10 numeric rating scale. The remaining two ASDD items are only administered to those 16 years of age and older and address impact and bothersomeness associated with axillary sweating (Items 3 and 4, respectively). Concurrent with the development of the ASDD, additional supportive assessments were included for use in patients ≥16 years of age as follows: six items to assess the impact of hyperhidrosis on a weekly basis (Weekly Impact items; Table 1) and the single-item PGIC to assess overall change in hyperhidrosis severity over the study course (Table 1). As described above, a child-specific, 2-item version of the ASDD (ASDD-C; Table 1) was similarly developed for use in patients ≥9 to < 16 years of age.

### Psychometric evaluation

The phase 2 and phase 3 patient pools were similar (Table 3) with the exception that the phase 3 trials allowed for the inclusion of younger patients. Specifically, 32 patients that were ≥ 9 to < 16 years of age (average age 13.8 years) were included in the phase 3 studies and administered the 2-item ASDD-C.

**Table 3:** Patient Demographic and Hyperhidrosis History Data From Phase 2 and Phase 3 Trials

Characteristic	Phase 2: DRM04-HH02 (*N* = 105)		Phase 3 Pooled: ATMOS-1 & ATMOS-2	
			Age − 9-15 Years (*N* = 32)	Age ≥ 16 Years (*N* = 665)
Age (years), mean ± SD	33.3 ± 11.7	32.7 ± 11.4	13.8 ± 1.37	33.6 ± 10.9
Axillary hyperhidrosis history (years), mean ± SD	NA	15.5 ± 10.8	3.4 ± 2.77	16.1 ± 10.7
Female, n (%)	48 (45.7)	371 (53.2)	27 (84.4%)	344 (51.7)
White, n (%)	91 (86.7)	570 (81.8)	26 (81.3%)	544 (81.8)
Weekly Impact Items, n (%) responding Yes to each statement				
Change shirt during the day	79 (75.2%)		N/A	517 (77.7%)
Take more than 1 shower or bath during the day	58 (55.2%)		N/A	345 (51.9%)
Feel less confident	84 (80.0%)		N/A	551 (82.9%)
Feel embarrassed	88 (83.8%)		N/A	583 (87.7%)
Avoid interactions with others	51 (48.6%)		N/A	402 (60.5%)
Prevent from doing activities	43 (41.0%)		N/A	352 (52.9%)
Weekly Impact Summary,^a^ mean ± SD	3.8 ± 1.90		N/A	4.1 ± 1.90

*N/A* Not available, data not collected during the study course, *SD* Standard deviation

^a^Summary score is derived by assigning a score of 1 for each “Yes” response (range 0 to 6). Validity of the Weekly Impact Summary score has not been tested via dimensionality analyses and should be considered exploratory at this stage

#### Descriptive statistics and reliability

No floor/ceiling effects were identified for the ASDD/ASDD-C axillary sweating severity (Item 2) and impact (Item 3) items; there was some ceiling effect (21.4%) for the bothersomeness item (Item 4; Table 4), which indicated that approximately one fifth of patients were extremely bothered by their condition at baseline. The ASDD also demonstrated adequate test-retest reliability between Week 3 and 4 (ICCs ~ 0.9 for Items 2–4), well above the 0.70 threshold.

**Table 4:** ASDD/ASDD-C Measurement Properties Evaluated in Phase 2 and Phase 3 Trials

Measurement Property			Phase 2: DRM04-HH02 (*N* = 105)	Phase 3 Pool: ATMOS-1 & ATMOS-2	
				Age 9–15 Years (*N* = 32)	Age ≥ 16 Years (*N* = 665)
Mean ± SD [median] Floor and Ceiling Effects, Non-response Bias, N(%)	Axillary Sweating Severity (Item 2)	Baseline^a^	6.8 ± 1.9 [7.0]	6.8 ± 2.5 [7.0]	7.2 ± 1.6 [7.4]
		Minimum (0)	3 (2.9%)	0 (0%)	1 (0.2%)
		Maximum (10)	9 (8.6%)	6 (18.8%)	83 (12.5%)
	Impact (Item 3)	Baseline^a^	2.2 ± 0.9 [2.2]	N/A	2.4 ± 0.9 [2.3]
		Minimum (0)	12 (11.4%)		31 (4.7%)
		Maximum (4)	10 (9.5%)		105 (15.8%)
	Bothersomeness (Item 4)	Baseline^a^	2.3 ± 0.9 [2.3]	N/A	2.6 ± 0.9 [2.6]
		Minimum (0)	11 (10.5%)		13 (2.0%)
		Maximum (4)	15 (14.3%)		145 (21.8%)
Test-Retest Reliability, ICC	Axillary Sweating Severity (Item 2)	Week 4 – Week 3	0.91	0.92	0.94
	Impact (Item 3)	Week 4 – Week 3	0.89	N/A	0.90
	Bothersomeness (Item 4)	Week 4 – Week 3	0.88	N/A	0.89
Item 2 Responsiveness		Week 4 – Baseline			
Sweat Production,^b^ r^c^			0.21^f^	0.01^d,e^	0.22^d,e^
Effect Size of Change (SD Baseline units)			−2.2	−2.0	− 2.4
Standardized Response Mean			−1.6	−0.70	−1.5

*ASDD* Axillary Sweating Daily Diary, *ICC* Intraclass correlation, *SD* Standard deviation

^a^ For subjects with Week 4 scores

^b^ Measured gravimetrically

^c^ Based on the change from Baseline to Week 4 in ASDD Item 2 scores and sweat production

^d^ Spearman correlation coefficient

^e^ Based on the change from Baseline to Week 4 in ASDD Item 2 scores and the natural logarithm of sweat production

^f^ Pearson correlation coefficient

#### Construct validity

The pattern of correlations between ASDD items and other efficacy measures is supportive of the construct validity of the ASDD axillary sweating severity item (Item 2; Table 5). Specifically, strong positive correlations (*r* ≥ 0.5) were observed between ASDD Item 2 and HDSS grade at Week 4, as well as between the changes in both measures from Baseline. As expected, ASDD Item 2 was moderately to strongly correlated with the DLQI/C-DLQI (*r* = 0.61 /0.51 at Week 4 and *r* = 0.63/0.51 between changes in both measures from Baseline). Correlations between ASDD Item 2 and sweat production were weak to moderate at Week 4 (*r* = 0.17 to 0.33) and were weak for the changes in both measures from Baseline (*r* = 0.17 to 0.18), which was expected given the differences in reference period for these measures (ie, ASDD Item 2 daily assessments were averaged over a 7-day period whereas sweat production was assessed at a single point in time, once each week). Similar trends were noted for both the adult and pediatric populations in the phase 3 study. Exploratory analyses to evaluate the relationship between all newly developed items (ASDD, Weekly Impact, PGIC) showed strong relationships across the different measures. ASDD inter-item correlations were all > 0.80. While severity, impact, and bothersomeness in the ASDD may represent different constructs of hyperhidrosis, they are likely to be strongly associated with one another. Furthermore, ASDD Item 2 was strongly correlated with the Weekly Impact summary. In addition, the correlation between the change from Baseline in ASDD Item 2 was strongly correlated with change from Baseline in the Weekly Impact summary and the PGIC at Week 4 (Table 5). Finally, ASDD items addressing the impact and burden of sweating (Items 3 and 4, respectively) demonstrated a similar pattern of correlations (strong correlations with HDSS and Weekly Impact summary, moderate to strong correlation with DLQI, and a weak correlation with sweat production), though results were mixed with respect to Items 3 and 4 correlation with PGIC for the phase 2 and phase 3 data sets, with stronger correlations observed in the phase 3 set **(**Table 5).

**Table 5:** ASDD Construct Validity Correlations at Week 4 (Phase 2 data; *N* = 105 or Phase 3 data; *N* = 697)

ASDD Item		HDSS Grade, Pearson correlation	Sweat Production,^a^ Pearson correlation	DLQI/C-DLQI, Spearman correlation	Weekly Impact Summary, Pearson correlation	PGIC, Spearman correlation
Week 4 Average						
Axillary Sweating Severity (Item 2)	Phase 2	0.73*	0.33*	N/A	0.52*	0.62*
	Phase 3 9–15yo	0.71*	0.17	0.51*	N/A	N/A
	Phase 3 ≥ 16yo	0.70*	0.18*	0.61*	0.66*	0.70*
Impact (Item 3)	Phase 2	0.71*	0.24*	N/A	0.62*	0.65*^b^
	Phase 3 Pooled	0.72*	0.13*	0.65*	0.68*	0.65*
Bothersomeness (Item 4)	Phase 2	0.79*	0.27*	–	0.64*	0.70*^b^
	Phase 3 Pooled	0.74*	0.13*	0.66*	0.69*	0.66*
Change from Baseline to Week 4						
Axillary Sweating Severity (Item 2)	Phase 2	0.57*	0.17	N/A	0.65*	0.48*
	Phase 3 9–15yo	0.71*	0.17	0.51*	N/A	N/A
	Phase 3 ≥ 16yo	0.70*	0.18	0.63*	0.62*	0.68*
Impact (Item 3)	Phase 2	0.51*	0.05	N/A	0.57*	0.27*
	Phase 3 Pooled	0.64*	0.01	0.51*	0.65*	0.57*
Bothersomeness (Item 4)	Phase 2	0.58*	0.13	N/A	0.61*	0.31*
	Phase 3 Pooled	0.65*	0.02	0.53*	0.65*	0.59*

*ASDD* Axillary Sweating Daily Diary, *HDSS* Hyperhidrosis Disease Severity Scale, *PGIC* Patient Global Impression of Change

*p ≤ 0.05

^a^ Measured gravimetrically

^b^ Weekly average for PGIC is not applicable

Entries marked N/A indicate that the assessment was not performed for that study and/or population

#### Known-groups validity

Statistically significant differences in mean ASDD Item 2 scores and HDSS grade strata (*p* < 0.0001), gravimetrically-measured sweat production quartile (*p* = 0.009 for phase 2; *p* < 0.001 for phase 3) at Week 4, and DLQI (*p* < 0.0001) (Table 6) support the known-groups validity of ASDD Item 2 in the adult population. Similar trends were noted in the phase 3 pediatric population for HDSS Scores. While ASDD Item 2 scores were higher (more severe patient-reported sweating) for patients with greater sweat production (4.1 vs 6.3), the difference was not statistically significant, which could in part be due to the smaller sample size in the pediatric population (*n* = 32). Only 1 pediatric patient reported a very large to extremely large HRQOL impact due their skin condition on the C-DLQI; this patient reported a mean weekly ASDD score of 9.8 compared to 4.4 in the group with minimal HRQOL impact. Although no specific predictions were preplanned for ASDD Items 3 (impact) and 4 (bother), data in Table 6 show that these items were also able to discriminate between known groups.

**Table 6:** Known-Groups Validity – ASDD by HDSS, Gravimetry, and DLQI/CDLQI severity thresholds at Week 4

		Phase 2: DRM04-HH02 (*N* = 105)	Phase 3 Pooled: ATMOS-1 & ATMOS-2	
			Age 9–15 Years (*N* = 32)	Age ≥ 16 Years (*N* = 665)
ASDD Item 2 (Week 4 Average)	HDSS, mean (SD)			
	Grade 1	0.9 ± 1.2 (*n* = 35)	2.0 ± 0.6 (*n* = 5)	1.1 ± 1.6 (*n* = 208)
	Grade ≥ 3	5.3 ± 1.4 (*n* = 14)	6.8 ± 1.86 (*n* = 13)	6.5 ± 2.1 (*n* = 154)
	*p*-value	< 0.001	< 0.001	< 0.001
	Sweat Production,^a^ mean (SD)			
	1st Quartile	1.8 ± 1.7 (*n* = 19)	4.1 ± 3.2 (*n* = 3)	2.6 ± 2.6 (*n* = 166)
	4th Quartile	3.4 ± 2.0 (*n* = 20)	6.3 ± 1.9 (*n* = 15)	4.2 ± 2.8 (*n* = 168)
	*p*-value	0.009	0.125	< 0.001
	DLQI/C-DLQI, mean (SD)			
	No to moderate impact	N/A	4.4 ± 2.5 (*n* = 27)	2.9 ± 2.5 (*n* = 571)
	Very large to extremely large impact	N/A	9.8 (*n* = 1)	7.3 ± 2.4 (*n* = 7)
	*p*-value	N/A	N/A^b^	< 0.001
ASDD Item 3 (Week 4 Average)	HDSS, mean (SD)			
	Grade 1	0.2 ± 0.4 (*n* = 35)	N/A	0.2 ± 0.3 (*n* = 182)
	Grade ≥ 3	1.5 ± 0.7 (*n* = 14)	N/A	2.1 ± 1.0 (*n* = 114)
	*p*-value	< 0.001	N/A	< 0.001
	Sweat Production,^a^ mean (SD)			
	1st Quartile	0.5 ± 0.6 (*n* = 19)	N/A	0.7 ± 0.8 (*n* = 146)
	4th Quartile	1.0 ± 0.7 (*n* = 20)	N/A	1.2 ± 1.0 (*n* = 134)
	*p* -value	0.026	N/A	< 0.001
	DLQI/C-DLQI, mean (SD)			
	No to moderate impact	N/A	N/A	0.8 ± 0.9 (*n* = 508)
	Very large to extremely large impact	N/A	N/A	3.0 ± 1.0 (n = 5)
	*p* -value	N/A	N/A	< 0.001
ASDD Item 4 (Week 4 Average)	HDSS, mean (SD)			
	Grade 1	0.2 ± 0.3 (*n* = 35)	N/A	0.2 ± 0.3 (*n* = 182)
	Grade ≥ 3	1.7 ± 0.7 (*n* = 14)	N/A	2.3 ± 1.0 (*n* = 114)
	*p* -value	< 0.001	N/A	< 0.001
	Sweat Production,^a^ mean (SD)			
	1st Quartile	0.5 ± 0.5 (*n* = 19)	N/A	0.8 ± 0.8 (*n* = 146)
	4th Quartile	1.2 ± 0.7 (*n* = 20)	N/A	1.3 ± 1.0 (*n* = 134)
	*p* -value	0.002	N/A	< 0.001
	DLQI/C-DLQI, mean (SD)			
	No to moderate impact	N/A	N/A	0.9 ± 0.9 (*n* = 508)
	Very large to extremely large impact	N/A	N/A	3.0 ± 1.1 (*n* = 5)
	*p* -value	N/A	N/A	< 0.001

*ASDD* Axillary Sweating Daily Diary, *HDSS* Hyperhidrosis Disease Severity Scale, *SD* Standard deviation

^a^ Measured gravimetrically; ^b^Not calculable due to sample size (*N* = 1)

*P* value for two sample t-test

HDSS Grade 1 = My sweating is never noticeable and never interferes with my daily activities; Grade 3 = My sweating is barely tolerable and frequently interferes with my daily activities; Grade 4 = My sweating is intolerable and always interferes with my daily activities

Quartiles for gravimetric measurement of sweat production at Week 4 defined as 25th percentile (8.7 mg/axilla/5 min) and 75th percentile (34.95 mg/axilla/5 min)

DLQI: no to moderate impact = range 0–10; large to extremely large impact = range 21–30; CDLQI: no to moderate impact = range 0–12; large to extremely large impact = range 19–20

#### Responsiveness

The responsiveness of ASDD/ASDD-C Item 2 (ability to detect change in sweating severity) was demonstrated by large effect sizes (ranging from − 2.2 to − 2.4), as well as by correlations that were within the expected range for the change in Item 2 and the change in the gravimetric measures of sweat production (Table 4). In addition, the correlation between the change from Baseline in ASDD Item 2 was strongly correlated with change from Baseline in the Weekly Impact summary and the PGIC at Week 4 (Table 5).

A PGIC rating of “moderately better” within this scale corresponded to a 3.8-point change on the ASDD Item 2 in both phase 2 and phase 3 studies (Table 7). A “moderately better” category was used to indicate a clinically meaningful change from the patient perspective. Patients achieving reductions in weekly average scores on ASDD Item 2 of ≥4 points were defined as responders to treatment. Within-person changes in HDSS scores against PGIC responses followed a similar trend as that of ASDD; similarly, within-person change scores on ASDD calculated across different magnitudes of change in HDSS scores showed that as HDSS scores improved, so did ASDD scores (Tables 7 and 8).

**Table 7:** Responder Estimates for ASDD Axillary Sweating Severity Item (Item 2): Anchor-Based Method (Phase 2 Data)

PGIC Response	Phase 2: DRM04-HH02 (*N* = 105)				Phase 3 Pooled: ATMOS-1 & ATMOS-2 (*N* = 697)			
	n (%)	ASDD Axillary Sweating Severity (Average Weekly Change), mean ± SD [median]	n (%)	HDSS change score (Average Weekly Change), mean ± SD [median]	n (%)	ASDD Axillary Sweating Severity (Average Weekly Change), mean ± SD [median]	n (%)	HDSS change score (Average Weekly Change), mean ± SD [median]
1 = Much better	50 (64.1)	− 5.1 ± 2.5 [− 5.5]	45	− 1.9 ± 0.80 [− 2.0]	276 (39.6)	−5.7 ± 2.05 [− 5.7]	276 (39.6)	− 2.0 ± 0.71 [− 2.0]
**2 = Moderately better**	**11 (14.1)**	**− 3.8 ± 2.7 [− 3.4]**	**10**	**− 1.5 ± 0.71 [− 1.0]**	**111 (15.9)**	**− 3.8 ± 2.28 [− 3.7]**	**111 (15.9)**	**− 1.4 ± 0.66 [− 1.0]**
3 = A little better	13 (16.7)	− 2.3 ± 1.2 [− 1.9]	14	− 0.6 ± 0.76 [0.0]	119 (17.1)	− 2.3 ± 1.97 [− 1.9]	119 (17.1)	− 0.9 ± 0.71 [− 1.0]
4 = No difference	4 (5.1)	− 1.7 ± 0.7 [− 1.9]	3	−0.3 ± 0.58 [0.0]	82 (11.8)	−1.0 ± 1.69 [−0.6]	82 (11.8)	−0.5 ± 0.74 [0.0]
5 = A little worse	0	–	0	–	11 (1.6)	0.2 ± 1.54 [0.3]	11 (1.6)	−0.4 ± 0.67 [0.0]
6 = Moderately worse	0	–	0	–	0	–	0	–
7 = Much worse	0	–	0	–	0	–	0	–

**Bold text** represents the preferred threshold for meaningful response consistent with FDA guidance (“moderately better”)

*ASDD* Axillary Sweating Daily Diary, *PGIC* Patient Global Impression of Change, *SD* Standard deviation

**Table 8:** ASDD Change Scores Across HDSS Improvements

HDSS Score	Phase 2: DRM04-HH02 (*N* = 105)		Phase 3 Pooled: ATMOS-1 & ATMOS-2 (*N* = 697)	
	n (%)	ASDD Axillary Sweating Severity (Average Weekly Change), mean ± SD [median]	n (%)	ASDD Axillary Sweating Severity (Average Weekly Change), mean ± SD [median]
3-point improvement	13 (16.7%)	−6.2 ± 2.68 [− 6.6]	74 (10.6)	−6.7 ± 2.29 [− 6.9]
2-point improvement	29 (37.2%)	− 5.2 ± 2.10 [− 5.3]	251 (36)	− 5.2 ± 2.13 [− 5.4]
1-point improvement	24 (30.8%)	−3.3 ± [− 3.2]	247 (35.4)	−2.9 ± 2.17 [− 3.0]
No improvement	12 (15.4%)	−1.9 ± 0.92 [− 2.0]	113 (16.2)	−0.9 ± 1.91 [− 0.5]
1-point worsening	0	–	12 (1.7)	−0.5 ± 1.47 [− 0.5]
2-point worsening	0	–	–	–
3-point worsening	0	–	–	–

## Discussion

The purpose of this research was to develop the ASDD and ASDD-C and demonstrate validity of Item 2, intended to measure severity of axillary sweating among patients with axillary hyperhidrosis in both adults and pediatric patients aged 9 and above. Qualitative and quantitative analyses presented here demonstrate strong relationships with other established outcomes in hyperhidrosis (construct and known-groups validity), good reproducibility (reliability), and establishment of a threshold indicating a meaningful magnitude of response via anchor-based methods (responsiveness) for use in evaluating axillary hyperhidrosis treatments in interventional studies. Validation analyses were highly consistent between the phase 2 and phase 3 studies, providing increased confidence in ASDD measurement properties and particularly the responder definition. This research highlights the importance of capturing the patient perspective for this condition, particularly in light of the limitations associated with more objective methods of assessing sweat production.

Given the level of unmet need among hyperhidrosis patients, novel assessment tools are needed to support future research and the development of new treatments. Although the HDSS has historically been used in the clinical trial setting to characterize patients with hyperhidrosis, it cannot be used in support of product approvals because patient input was not formally taken into consideration during its development. In addition, the HDSS combines two concepts within the same item (symptom severity and impact), limiting the ability to ascertain which of the two concepts has changed with treatment. The ASDD was developed in accordance with the recommendations outlined in the FDA PRO guidance to support U.S. product approvals [18]. This instrument was created based on patient input and clinical considerations, and the parallel development of a pediatric-specific version of the ASDD expands research capability across a range of patient populations. This novel PRO measure has added value in that ASDD/ASDD-C axillary sweating severity item (Item 2) can support efficacy assessments for product approvals and labeling, which may facilitate approval of new treatments for axillary hyperhidrosis. In addition, the results of qualitative and quantitative analysis for impact (Item 3) and bothersomeness (Item 4) support their inclusion as key endpoints in clinical trials. The Weekly Impact items were developed based on patient input to explore impacts on various areas of patients’ lives and offers valuable insight with regards to how patients cope with this condition on a regular basis. Additional research focusing on more distal impacts of the disease is warranted to further refine these items.

Several study limitations should be recognized. Foremost, the development of the ASDD/ASDD-C was based on a small, homogeneous patient population, which may not have reflected different levels of disease severity; additionally, the pediatric patients did not have a physician-confirmed diagnosis of hyperhidrosis for the qualitative development. Patients included in the phase 2 and phase 3 trials were also relatively homogeneous (ie, similar disease severity and demographic characteristics), potentially limiting generalizability of the measure. In addition, though the axillary sweating severity item is equivalent between the ASDD and ASDD-C, the cohort of patients ≥9 and < 16 years in the phase 3 trials was small. Despite this limitation, ASDD-C data in the pediatric population demonstrate results broadly consistent with those of adults. The focus of this research was primarily on the ASDD Item 2; while exploratory analyses showed that all the newly developed items demonstrated consistency with one another, dimensionality was not formally assessed to better understand their relationship with one another and whether it would be appropriate to derive summary scores. It also should be noted that initial draft items pertaining to severity were developed before patient engagement, which as a matter of process may be disadvantageous when seeking to identify constructs directly germane to a patient; however, for this hyperhidrosis measure, the concept of sweating severity was the focus of the study (Item 2) and was considered to be both straightforward and unambiguously relevant.

## Conclusions

Taken together, the rigorous development and validation of ASDD/ASDD-C axillary sweating severity item support its use in the evaluation of axillary hyperhidrosis treatment in clinical trials. Strong qualitative and quantitative evidence also supports the use of the impact and bothersomeness items as key trial endpoints in adult trials. Further evaluation of this measure in future research studies is warranted to demonstrate consistent performance across different axillary hyperhidrosis populations and in different study contexts.

Familiarity with the ASDD/ASDD-C along with the Weekly Impact items among clinicians who treat patients with hyperhidrosis may facilitate a better understanding of the impact of this condition on patients’ lives.

## Data Availability

The data that support the findings of this study are available from Dermira, Inc., but restrictions apply to the availability of these data, which were used under license for the current study, and so are not publicly available. Data are however available from the authors upon reasonable request and with permission of Dermira.

## Abbreviations

AHPM: Axillary Hyperhidrosis Patient Measures
ASDD: Axillary Sweating Daily Diary
ASDD-C: Child-specific version of the ASDD
C-DLQI: Children’s Dermatology Life Quality Index
DLQI: Dermatology Life Quality Index
GT: Glycopyrronium tosylate
HDSS: Hyperhidrosis Disease Severity Scale
HHIQ: Hyperhidrosis Impact Questionnaire
HidroQOL: Hyperhidrosis Quality of Life Index
HRQOL: Health-related quality of life
NHP: Nottingham Health Profile
PGIC: Patient Global Impression of Change
PRO: Patient-reported outcome
SD: Standard deviation
SF-36 and SF-12: Short Form Health Surveys
